# Occupational immediate type allergy to soapnut and quillaja bark 

**DOI:** 10.5414/ALX02131E

**Published:** 2021-01-26

**Authors:** Rolf Merget, Monika Raulf, Ingrid Sander

**Affiliations:** Institute for Prevention und Occupational Medicine of the German Social Accident Insurance, Institute of the Ruhr University Bochum (IPA), Germany

**Keywords:** allergy, asthma, occupational, quillaja bark, saponin, soapnut

## Abstract

A 58-year-old non-atopic chemical worker complained about work-related asthma and rhinoconjunctivitis about 4 years after exposure to quillaja bark and soapnut. Bronchial hyperresponsiveness was demonstrated after withdrawal of medication for 12 hours. Skin prick tests with extracts from quillaja bark and soapnut from the workplace were positive, but ImmunoCAP was positive only with quillaja bark, probably due to the low protein content of the extract from soapnut. Sensitizations to quillaja bark and soapnut, but not to saponin were demonstrated by immunoblot. An inhalation test with a dosimeter was positive with the soapnut extract. A link between disease and exposure was documented by serial measurements of exhaled nitric oxide at and off work, despite preventive measures. A diagnosis of occupational allergy due to quillaja bark and soapnut was made. Further exposure reduction was recommended.

**German version published in Allergologie, Vol. 43, No. 3/2020, pp. 98-103**

## Introduction 

The discovery of a new immediate-type occupational allergen with evidence of an IgE-mediated mechanism is a rare event. In this report, a yet unknown occupational allergen causing occupational asthma was identified, and a recommendation for recognition of an occupational disease was given to the compensation board. A case history of a worker with an immediate-type allergy to quillaja bark was described as early as 1980 [1], which was reproduced in this case study. An allergy to soapnut has not been reported so far, however, we were unable to identify the allergen in detail. 

## Case history 

A 58-year-old worker was referred to our institute with the question of a work-related obstructive airway disease. He spent his childhood and adolescence in Kazakhstan and was without any contact to hazardous substances until he moved to Germany. In Germany, he worked as a caretaker for about a year and then worked for 8 years in a small chemical company. He developed asthmatic complaints ~ 4 years after starting this job. He consulted a pneumologist for the first time ~ 3 years after the appearance of symptoms. The pneumologist made a diagnosis of asthma and reported his suspicion of occupational asthma to the compensation board according to the clearly work-related symptoms. The occupational hygienist of the board reported that the worker carried out various tasks in the company and had regular contact with natural raw materials as starting substances for the production of saponins. These were pressed oil fruits from India and quillaja bark from South America. The oil fruits (soapnuts) were delivered pressed (so-called mocha, saponin crude) and ground by the worker. Quillaja bark was delivered in bales, and stacked and occasionally cut by the worker. Due to his respiratory problems, preventive measures were successively initiated, but even an airstream helmet could not prevent persisting complaints. He was finally transferred internally to a department with ongoing but much less contact with natural substances about a year before the examination in our institute (IPA). 

At his first presentation in our institute, less than a year after the report of the suspected occupational disease, the worker confirmed work-related asthmatic and rhinoconjunctivitis complaints with no symptoms during holidays. There were no respiratory diseases or allergies before starting work in the saponin production. However, respiratory problems at work continued to occur at least once a week even after internal transfer. Between these events, the worker had no complaints. He inhaled steroids (ICS) and long-acting β-agonists (LABA) daily, the additional administration of short-acting β-agonists (SABA) was only rarely necessary. He suspected the raw materials for saponin production to be the trigger of his disease. He was an ex-smoker with ~ 10 pack years, but he was not atopic. Apart from mild arterial hypertension and chronic back pain, no other diseases were remembered. He gave informed consent to publish his case in a medical journal. 

## Methods and results 

### Basic examinations 

The physical examination of the patient was without pathological findings, with the exception of nasal obstruction. Basic blood analyses showed eosinophilia of 6%, but were otherwise normal. An electrocardiogram was normal. Lung function under anti-obstructive medication showed borderline obstruction with an FEV_1_ of 100% of the European Commission of Coal and Steel (ECCS) reference and an FEV_1_/FVC of 68%. Methacholine testing (reservoir method) showed severe bronchial hyperresponsiveness with a PD_20_FEV_1_ of 47 µg (the cut-off for bronchial hyperresponsiveness with this method is 300 µg) when medication was paused on the day of examination. Spiroergometry did not show pulmonary limitation at a normal exercise capacity. 

### Special examinations 

Skin prick tests (SPT) with a battery of common environmental allergens and molds (various manufacturers) were negative. The total IgE of 38 kU/L was in the normal range. SPT with samples from the patient’s workplace using extracts of medium finely ground soapnut raw material (saponin crude) at a protein concentration of 0.125 mg/mL and 0.5 mg/mL each showed a positive immediate reaction. The wheal and erythema diameters were 3 mm and 5 mm, respectively. At a protein concentration of 0.13 mg/mL, the quillaja bark extract led to a wheal diameter of 7 mm and an erythema diameter of 15 mm. No reaction could be observed with the final product saponin (concentration 35 µg/mL protein, produced from quillaja bark). 

For specific IgE determination, extracts of quillaja bark and soapnut raw material were bound to streptavidin ImmunoCAPs after biotinylation [2]. The saponin end product did not contain enough protein for coupling to the solid phase, and also the biotinylated soapnut extract had only a low protein concentration due to high losses caused by poor solubility during gel filtration to remove free biotin reagent. Therefore, no inhibition experiments were performed on the question of cross-reactivities between quillaja bark and soapnut. Elevated IgE antibodies could only be detected in the patient’s serum to quillaja bark at a concentration of 1.16 kU_A_/L (CAP class 2), but not to the soapnut extract. 

**Figure 1.**
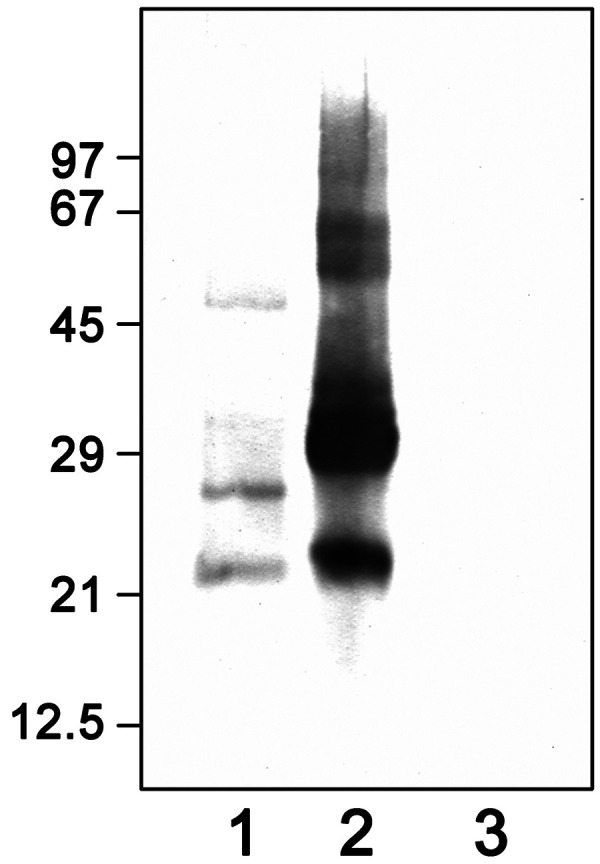
IgE immunoblot with the patient’s serum (molecular weights in kDa). Lane 1: quillaja bark; Lane 2: soapnut raw material; Lane 3: saponin (end product).

In order to identify IgE-binding components in the extracts, IgE-immonoblots were performed with the three extracts and the patient’s serum. In the extract from quillaja bark, 2 weak bands were detected at 21 and 27 kDa. In the soapnut raw material extracted with 0.05 M NaOH, proteins at 22 and 32 kDa reacted very strongly with the IgE antibodies of the patient, the saponin end product did not react (Figure 1). To prove the specificity, 9 control sera in addition to the patient’s serum were examined in another immunoblot with the soapnut raw material. One of the sera belonged to a person sensitized to gum arabic, whose case report has been published [3]. Only the patient serum reacted in the immunoblot (data not shown). 

An extract from purchased soapnuts (*Sapindus trifoliatus*; Henschke, Mudenbach, Germany) showed a different band pattern in gel electrophoresis than the soapnut material from the patient’s workplace (data not shown). 

**Figure 2.**
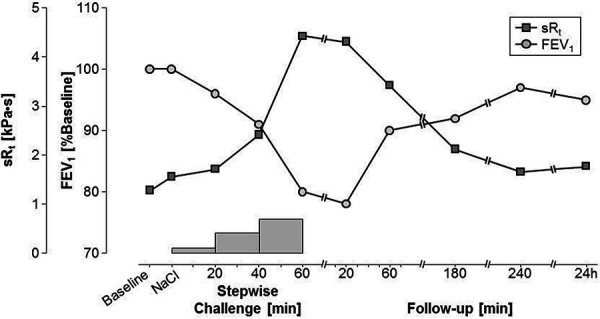
Time-response curve for specific airway resistance (sR_t_) and FEV_1_ of the inhalation test with a soapnut extract from the patient’s workplace. The maximum cumulative inhaled dose was ~ 4 µg protein.

A specific inhalation test was performed with an APSpro dosimeter and a DeVilbiss 646 nebulizer (Jäger, Würzburg, Germany). We used an extract of soapnut raw material with an initial concentration of 2 µg/mL protein (dose 176 ng protein) and increased in four-fold steps. After the third step (concentration 30 µg/mL; cumulative dose ~ 4 µg), the patient developed shortness of breath, FEV_1_ dropped to a minimum of 77% of the initial value 20 minutes after inhalation, and the specific respiratory resistance rose from 1.27 kPa.s to 4.3 kPa.s at this point (Figure 2). As both the spirometric and the bodyplethysmographic positivity criteria were fulfilled, the test was terminated. Without intervention, the airway obstruction almost completely disappeared within the following 4 hours, and no late reaction occurred. Exhaled nitric oxide (FeNO) increased slightly from 36 ppb to 44 ppb, and bronchial hyperresponsiveness was unchanged 24 hours after specific inhalation testing (PD_20_FEV_1_ 47 µg methacholine at baseline vs. 64 µg post challenge). 

**Figure 3.**
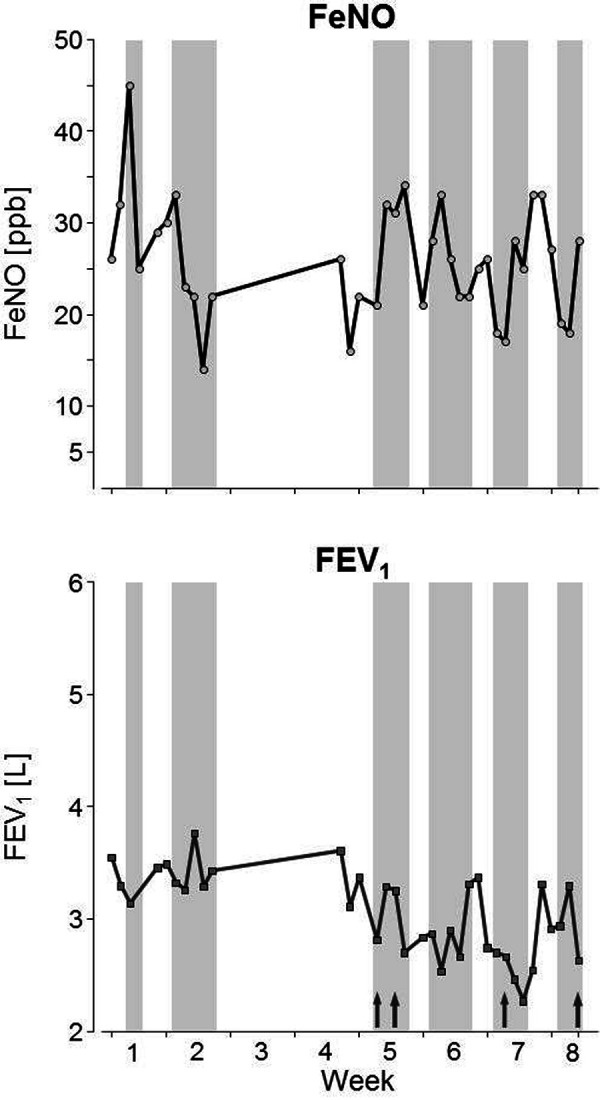
Serial measurements of exhaled nitric oxide FeNO (upper part) and FEV_1_ (lower part) during working periods (gray areas) and holidays or weekends. Arrows indicate medication with short-acting β-agonists.

Serial measurements of FEV_1_ and FeNO during work periods and a holiday were carried out once a day by the patient for almost 2 months, but no measurements were taken during a 2 weeks holiday in Kazakhstan (Figure 3). During working periods, the patient repeatedly documented exposure to raw materials. It was shown that FeNO increased during work periods, whereas FEV_1_ decreased. Asthma medication on demand with SABA was administered on a few working days, but not during holidays. 

## Discussion 

Saponins are potent surface-active agents that have a wide range of applications due to their foam-forming properties in aqueous solutions. They are added to beer, for example, or used in pharmaceuticals, but above all they have been used for centuries as body cleansers and detergents. Saponins are mainly extracted from quillaja bark *(Quillaja saponaria)* from Chile, Peru, or Brazil as well as from soapnuts from Asia (mainly India). The latter are also increasingly used as “eco-detergents” for textile cleaning. Soapnuts are the dried fruits of trees that belong to the soapberry family (Sapindaceae). The shell of the approximately hazelnut-sized “nuts” contains up to 15% saponins. There are two Indian soapnut species: the larger soapnut from *Sapindus mukorossi* and the smaller soapnut from *Sapindus trifoliatus*, which grows mainly in southern India. According to an internet search, the soapnut processed in the patient’s company probably came from a plantation in southern India, so that it initially appeared to be the smaller soapnut from *Sapindus trifoliatus*. The patient’s employer did not provide any information on the origin of the soapnuts. However, experiments with soapnuts of the species *Sapindus trifoliatus* showed a different band pattern in SDS-polyacrylamide gel electrophoresis than the material from the workplace and also no specific IgE-binding in the immunoblot, so that the sensitization of the patient is probably not due to *Sapindus trifoliatus*. Thus, the precise nature of the allergen in this case remains unknown. 

We demonstrated sensitization of the patient to quillaja bark by SPT and specific IgE using ImmunoCAP. Specific bands (21 and 27 kDa) were also found in the IgE immunoblot with the patient’s serum. 

An immediate-type allergy to quillaja bark had already been published using in vitro tests and inhalation testing [1]. However, no immunoblot was carried out at that time, and it was not clear whether carbohydrate components were responsible for the reactivity due to IgE reactions to gum arabic and gum tragacanth. The saponins from quillaja bark and soapnuts contain carbohydrate components that are bound to steroids, steroid alkaloids, or triterpenes. In this study, neither the SPT nor the immunoblot showed a reaction with the saponin end product. On the other hand, in addition to sensitization to quillaja bark, there was also sensitization to soapnut as shown by SPT and immunoblot (specific bands at 22 and especially at 32 kDa), but not in the ImmunoCAP, probably due to the low solubility of the soapnut proteins. Therefore, ImmunoCAP inhibition experiments to clarify possible cross-reactivity of IgE antibodies against quillaja bark and soapnut were not carried out. Most likely, it is a co-sensitization to quillaja bark and soapnut. An inhalation challenge with the soapnut extract was clearly positive in the sense of an immediate type reaction. The absence of a conspicuous increase of FeNO or bronchial hyperresponsiveness after the specific inhalation test does not exclude an immunological mechanism, as the sensitivity of these additional methods is known to be low [4]. 

On the basis of the proven sensitization and the positive inhalation test with an occupational substance, we recommended recognizing the patient’s asthma as an occupational disease. Since the patient still reported work-related complaints after an internal transfer, it was suspected that the patient was still being exposed. In order to check whether he was still endangered, we initiated daily measurements of FeNO and FEV_1_ over a period of several weeks at work and during a holiday. No clear increase of FeNO during working periods was observed, but a decrease in FEV_1_ with occasional SABA medication. Thus, further preventive measures and, if not sufficient, complete exposure cessation were recommended. Unfortunately, the patient was lost to follow-up. 

## Acknowledgment 

We thank Mrs. Angelika Flagge and Mrs. Ursula Meurer for the excellent performance of the allergological in vitro tests. 

## Funding 

The study was financed by the German Social Accident Insurance (projects IPa-004 and IPA-14). 

## Conflict of interest 

The authors declare that there is no conflict of interest.
